# FSTL1 aggravates LPS-induced liver injury through the NLRP3-CASP1-GSDMD and BAX-CASP3-GSDME pyroptosis pathways

**DOI:** 10.3389/fimmu.2026.1984625

**Published:** 2026-10-30

**Authors:** Yu Duan, Zechen Shan, Renqi Yao, Yuguo Chen

**Affiliations:** 1 Department of Emergency Medicine, Qilu Hospital of Shandong University, Jinan, China; 2 Chest Pain Center, Shandong Provincial Clinical Research Center for Emergency and Critical Care Medicine, Institute of Emergency and Critical Care Medicine of Shandong University, Qilu Hospital of Shandong University, Jinan, China; 3 Key Laboratory of Emergency and Critical Care Medicine of Shandong Province, Key Laboratory of Cardiopulmonary-Cerebral Resuscitation Research of Shandong Province, Shandong Provincial Engineering Laboratory for Emergency and Critical Care Medicine, Qilu Hospital of Shandong University, Jinan, China; 4 The Key Laboratory of Cardiovascular Remodeling and Function Research, Chinese Ministry of Education, Chinese Ministry of Health and Chinese Academy of Medical Sciences, The State and Shandong Province Joint Key Laboratory of Translational Cardiovascular Medicine, Qilu Hospital of Shandong University, Jinan, China; 5 Medical Innovation Research Division and Fourth Medical Center of the Chinese PLA General Hospital, Beijing, China; 6 Department of General Surgery, The First Medical Center of the Chinese PLA General Hospital, Beijing, China

**Keywords:** FSTL1, inflammation, pyroptosis, ROS, septic liver injury

## Abstract

**Introduction:**

Hepatocellular pyroptosis and its proinflammatory effects critically contribute to the pathology of acute liver injury. FSTL1, a secreted glycoprotein, has been implicated in regulating multiple cell death pathways and promoting inflammatory disease progression. However, the regulatory mechanisms of FSTL1-mediated hepatocellular pyroptosis in sepsis-induced liver injury remain poorly understood. This study aimed to investigate the role of FSTL1 in hepatocyte pyroptosis during sepsis and evaluate its therapeutic potential.

**Methods:**

Septic liver injury was induced by intraperitoneal LPS injection in mice. Hepatocyte-specific FSTL1 knockdown was achieved via AAV-mediated gene silencing prior to LPS challenge. Serum and liver tissues were collected at designated time points. We measured serum FSTL1, liver function markers (AST, ALT), evaluated histopathological changes, ROS production, pyroptosis pathway activation (NLRP3-CASP1-GSDMD and BAX-CASP3-GSDME), hepatic inflammation, and survival rates.

**Results:**

Serum FSTL1 was significantly elevated and positively correlated with liver injury severity. Hepatic FSTL1 expression progressively increased during sepsis, closely paralleling disease progression. Both NLRP3-CASP1-GSDMD and BAX-CASP3-GSDME pyroptosis pathways were time-dependently activated in hepatocytes under septic conditions. Notably, hepatocyte-specific FSTL1 knockdown attenuated ROS production, suppressed both pyroptotic pathways, reduced hepatic inflammation and tissue damage, and improved survival in septic mice.

**Discussion:**

FSTL1 plays a critical role in promoting hepatocellular pyroptosis during sepsis-induced liver injury through dual activation of NLRP3-CASP1-GSDMD and BAX-CASP3-GSDME pathways. Targeting FSTL1 may represent a promising novel therapeutic strategy for sepsis management.

## Introduction

Sepsis is defined as life-threatening organ dysfunction caused by a dysregulated organismal response to infection (1, 2). Although significant advances have been made in elucidating the complex pathological mechanisms of sepsis and developing numerous effective treatments, its morbidity and mortality rates remain high (3–6). It has been proven that the liver is critically involved in the progression of sepsis due to its crucial role in regulating metabolic processes and immune defenses (7, 8). However, lethal inflammatory responses can trigger hepatocellular death and organ failure (9–11). Among septic patients, the mortality rate for liver dysfunction or failure is as high as 54-68% (7). Therefore, it is of great significance to develop new and effective therapeutic strategies for ameliorating sepsis-induced liver injury.

Pyroptosis is a form of inflammatory cell death that plays a vital role in various human diseases, including cancer, cardiovascular disease and sepsis (12–15). The Gasdermin protein family members, mainly gasdermin D (GSDMD) and GSDME, are the key executors in pyroptosis, and their activation relies on inflammatory caspases (16, 17). When cells are stimulated by bacteria or viruses, the inflammasome, such as NACHT, LRR and PYD domains-containing protein 3 (NLRP3), can induce caspase-1 (CASP1) activation. In turn, activated CASP1 cleaves GSDMD to produce GSDMD-N, which permeabilizes the plasma membrane and triggers pyroptosis (16). Similarly, the GSDME can be specifically cleaved by CASP3, a process that relies on activation of the classical apoptotic signaling pathway (17). Previous studies have demonstrated that pyroptosis is markedly upregulated in the livers of septic mice, and this form of programmed cell death (PCD) constitutes a major contributor to sepsis-induced liver injury and failure (18, 19). In contrast, pharmacological or genetic inhibition of hepatocyte pyroptosis has been shown to attenuate liver damage and improve survival rates in septic mice (18, 19). These findings suggest that targeted modulation of pyroptotic pathways could be a promising therapeutic strategy for sepsis-associated liver injury. Nevertheless, the precise molecular mechanisms regulating hepatocyte pyroptosis during sepsis remain incompletely understood.

It is widely accepted that follistatin-like protein 1 (FSTL1), a secreted glycoprotein, is highly expressed in the liver and plays a proinflammatory role in human diseases (20, 21). Emerging evidence has demonstrated that FSTL1 is critically involved in various PCD pathways, including apoptosis, pyroptosis, and ferroptosis. For example, Shen et al. found that FSTL1 can mediate SMAD family member 2/3 (Smad2/3) phosphorylation in a transforming growth factor beta (TGF-β)-dependent manner to promote nucleus pulposus-derived mesenchymal stem cell apoptosis in intervertebral disc degeneration (22). Under lipopolysaccharide (LPS) stimulation, FSTL1 was found to enhance the transcription of NLRP3 and CASP1 in macrophages, which further aggravates pyroptosis and promotes the release of inflammatory cytokines (23). Cancer-associated fibroblasts (CAFs) have been confirmed to accelerate natural killer (NK) cell ferroptosis by releasing FSTL1. Mechanistically, CAF-derived FSTL1 can upregulate the expression of nuclear receptor coactivator 4 (NCOA4) at the transcriptional level through Disco Interacting Protein 2 Homolog A (DIP2A)-P38 pathway, which in turn accelerates NK ferroptosis by promoting NCOA4-mediated ferritinophagy (24). Although the FSTL1-related cell death is intimately associated with human diseases, the potential role and its regulatory mechanism in sepsis remain rarely investigated.

Herein, we aimed to elucidate the role of FSTL1 in septic liver injury. Our study yielded three major findings. First, both serum FSTL1 levels and hepatic FSTL1 expression were progressively upregulated during sepsis progression and were significantly correlated with the severity of liver injury. Second, both the NLRP3-CASP1-GSDMD and Bcl-2-associated X protein (BAX)-CASP3-GSDME pyroptotic pathways were time-dependently activated in hepatocytes under septic conditions. Third, hepatocyte-specific FSTL1 knockdown was associated with reduced ROS production, attenuated GSDMD/GSDME-related cell-death signaling, and amelioration of LPS-induced liver injury. Moreover, in isolated primary hepatocytes, ROS scavenging with N-acetylcysteine (NAC) suppressed both pyroptotic pathways and reduced hepatocyte death, supporting ROS as a downstream mediator of FSTL1-driven pyroptosis. Collectively, these findings suggest that FSTL1 contributes to LPS-induced septic liver injury at least in part through ROS-mediated pyroptosis and identify FSTL1 as a potential biomarker candidate and therapeutic target warranting further validation in polymicrobial sepsis models and clinical studies.

## Materials and methods

### Animal experimental model

WT C57BL/6J mice aged 6–8 weeks (20–25 g) were purchased from the Laboratory Animal Science of Chinese Academy of Medical Sciences, Beijing, China. All experimental procedures were conducted in accordance with the National Institutes of Health Guide for the Care and Use of Laboratory Animals and were approved by the Scientific Investigation Board of the Chinese PLA General Hospital (No. 2024-X20-40). Two independent cohorts were used. For LPS time-course experiments, mice were purchased at 6–8 weeks of age and used directly. For AAV8-mediated FSTL1 knockdown experiments, mice were purchased at 4 weeks of age. AAV8-TBG-ShFSTL1 or AAV8-TBG-ShCtrl (Genechem, Shanghai, China) was injected via the tail vein at 2 × 10¹¹ vector genomes per mouse at 4 weeks of age. The shRNA targeting mouse FSTL1 was 5’-AGGCCTGTGTGTGGCAGTAAT-3’, and the scrambled control sequence was 5’-GTAGCGCGGTGTATTATAC-3’. Hepatic FSTL1 knockdown was verified at 6 weeks of age in a separate validation cohort.

For the LPS time-course experiments, mice (6–8 weeks old) were randomly assigned to experimental groups and sacrificed at 6, 12, and 24 h after intraperitoneal injection of LPS (E. coli O111:B4, Sigma-Aldrich, L2630) at 10 mg/kg (n = 3 per group per time point). The same LPS dose (10 mg/kg) was used in all subsequent experiments. For AAV intervention experiments, AAV-injected mice received LPS at 8 weeks of age (2 weeks after AAV injection)(n = 3 per group), and FSTL1 knockdown was re-confirmed at the experimental endpoint by Western blot. Successful model establishment was confirmed by reduced body temperature and activity, elevated serum ALT/AST, increased serum inflammatory cytokines (TNF-α, IL-6, IL-1β), and characteristic hepatic histopathological injury. For the survival experiment, AAV-injected mice (ShCtrl or ShFSTL1) were randomly assigned to four groups: ShCtrl-con, ShFSTL1-con, ShCtrl-LPS, and ShFSTL1-LPS (n = 20 per group). Mice in the LPS groups received a single intraperitoneal injection of LPS, while mice in the control groups received an equal volume of saline. Survival was monitored every 6–12 h for 7 days. Mice were euthanized upon reaching any of the following humane endpoints: body weight loss >20%, severe lethargy or inability to move, hunched posture, labored breathing, or inability to access food/water.

### Biochemical analysis

FSTL1, IL-1β, IL-6, IL-18, LDH, HMGB1 and TNF-α were measured using ELISA kits according to the manufacturer’s instructions. The catalog numbers were as follows: FSTL1 (Abcam, ab316895), IL-1β (Abcam, ab197742), IL-6 (Abcam, ab222503), IL-18 (Abcam, ab218808), LDH (Abcam, ab102526), HMGB1 (Beyotime, PH406) and TNF-α (Abcam, ab208348). Serum alanine aminotransferase (ALT) and aspartate aminotransferase (AST) levels were measured using an automated chemical analyzer (BS-240 Chemistry Analyzer; Mindray, Shenzhen, China).

### Western blot analysis

For protein extraction, cells were lysed in RIPA lysis buffer (Huaxingbio, HX1862-1) supplemented with protease and phosphatase inhibitor cocktail (Thermo Fisher, 78440) and incubated on ice for 30 min. Liver tissues were homogenized in ice-cold RIPA lysis buffer containing protease and phosphatase inhibitors using a tissue homogenizer at a ratio of 1:10 (w/v), followed by incubation on ice for 30 min. Lysates were then centrifuged at 12,000 × g for 15 min at 4 °C, and the supernatants were collected. Protein concentration was determined using a BCA protein assay kit (Beyotime, P0012S) according to the manufacturer’s instructions. Equal amounts of total protein (20–30 µg per lane) were loaded and separated by SDS-PAGE on 12% gels, and proteins were transferred to polyvinylidene fluoride membranes with a pore size of 0.45 µm under constant current 200 mA for 1.5 h at 4 °C. The membranes were blocked and incubated with primary antibodies against FSTL1 (1:1000, Proteintech, 20182-1-AP), NLRP3 (1:1000, CST, 15101T), BAX (1:1000, CST, 2772T), GSDMD (1:1000, Abcam, ab209845), GSDME (1:1000, Abcam, ab215191), CASP1 (1:1000, Abcam, ab179515), CASP3 (1:1000, CST, 9661T), and β-actin (1:1000, Abcam, ab8226), followed by HRP-conjugated anti-mouse (1:5000, Proteintech, SA00001-1) or anti-rabbit (1:5000, Proteintech, SA00001-2) secondary antibodies. Equal total protein loading was confirmed before β-actin normalization, and protein expression levels were standardized to β-actin. Samples showing evident protein degradation (e.g., smeared or absent β-actin bands) were excluded from analysis.

### Histological examination

The dissected liver tissues were fixed in 4% paraformaldehyde (PFA) overnight at 4 °C and subsequently embedded in paraffin blocks. Tissue sections (4–5 μm thick) were deparaffinized and stained with hematoxylin-eosin (HE). Histological features were observed and analyzed using a microscope (Nikon Instruments Co., Japan). Two experienced histologists, blinded to the experimental groups, independently evaluated the sections. Hepatic injury was scored using a semi-quantitative system adapted from Suzuki’s criteria, assessing five parameters: hepatocyte vacuolation, sinusoidal congestion, inflammatory infiltration, hepatocyte necrosis, and hemorrhage. Each parameter was graded from 0 to 4 as follows: 0, none; 1, mild (<30% of the field); 2, moderate (30%–60%); 3, severe (>60%); and 4, very severe (>60% with structural disruption). Five randomly selected fields per section were evaluated at ×400 magnification. The scores from each parameter were summed to obtain a total hepatic injury score, with higher scores indicating more severe injury. The scoring system was adapted from Suzuki et al (25).

### Flow cytometry analysis

To assess hepatocyte death, cells were resuspended in 200 μL PBS and incubated with 5 μL PI (Biolegend, 640914) for 15 min at room temperature in the dark. For ROS detection, hepatocytes were incubated with the DHE fluorescence probe (Beyotime Biotechnology, S0063; 10 μM) for 10 min at room temperature in the dark. After incubation, cells were washed and resuspended in PBS for flow cytometry. For PI analysis, positive cells were identified using unstained and single-stained controls, and the percentage of PI^+^ cells was calculated. For DHE analysis, the mean fluorescence intensity (MFI) in the PE channel was determined after gating on hepatocytes, with unstained cells used to set the baseline. At least 10,000 events were collected per sample. Data were acquired using FACSDiva V 7.0 software (BD Biosciences, Mountain View, CA) and analyzed using FlowJo Version 10.0 software.

### ROS detection

Liver tissues were embedded in OCT compound and snap-frozen, and 5–10 μm frozen sections were prepared. The sections were rehydrated in PBS for 5 min and then incubated with DHE fluorescence probe (Beyotime Biotechnology, S0063; 10 μM in PBS) for 30 min at 37 °C in the dark. After incubation, the sections were washed three times with PBS (5 min each) and mounted with anti-fade mounting medium. Images were captured using a laser scanning confocal microscope (Leica, Mannheim, Germany) with a 20× objective. DHE oxidation was detected in the red channel with an excitation wavelength of 535 nm and an emission wavelength of 610 nm. All images were acquired under identical settings (laser power, gain, and offset) across groups.

### Cell isolation, culture and treatment

The isolation of primary hepatocytes was conducted as previously described. In brief, the needle was inserted into the mouse inferior vena cava, and the liver was perfused to wash out blood and circulating cells with warmed Hank’s balanced salt solution (HBSS, without calcium or magnesium, Gibco) containing 1% penicillin streptomycin. Then, DMEM/F12 (Gibco) containing 0.05% collagenase IV (Gibco) and 1% penicillin streptomycin was perfused to the liver to digest collagen in the extracellular matrix, thus accelerating cell dispersion. Next, the liver was dissected out and placed into a 6 cm culture dish with 10 mL DMEM/F12. The liver capsule was torn with forceps, and the liver was gently shaken to release the cells. Finally, the cell suspension was filtered through a 70 μm cell strainer and centrifuged at 50 × g for 3 min at 4 °C. Hepatocytes were in the pellet. These freshly isolated primary hepatocytes were used for subsequent experiments, including LPS stimulation, ROS detection, flow cytometric analysis, Western blot, and ELISA. Additionally, primary mouse hepatocytes were treated with LPS (100 ng/mL) in the presence or absence of the ROS scavenger *N*-acetylcysteine (NAC) (10 mM) for 24 h. Cells treated with an equal volume of PBS were used as controls. After treatment, cells and culture supernatants were collected for subsequent analyses.

### Statistical analysis

All data are presented as mean ± standard deviation (SD). For comparisons among multiple groups, one-way ANOVA followed by Bonferroni *post hoc* correction was applied. Spearman’s rank correlation coefficient was used to evaluate correlations between variables. Survival curves were generated using the Kaplan-Meier method, and differences between groups were assessed using the log-rank test. All statistical analyses were performed using SPSS software version 23.0 (SPSS Inc., Chicago, IL) and GraphPad Prism 8. A two-tailed P value < 0.05 was considered statistically significant (*P < 0.05; ns = not significant).

## Results

### Serum FSTL1 is positively associated with septic liver injury

To explore the role of FSTL1 in septic liver injury, we conducted an LPS-induced sepsis model by injecting LPS intraperitoneally. We detected the serum levels of FSTL1, aspartate aminotransferase (AST), and alanine aminotransferase (ALT) in septic mice at different time points (0, 6, 12, and 24 h). The results revealed that the levels of FSTL1, AST, and ALT gradually increased as sepsis developed (**Figures 1A–C**). The correlation analysis found that circulating FSTL1 level had a positive correlation with AST and ALT concentrations (**Figure 1D, E**). These findings indicated a strong connection between FSTL1 and septic liver injury.

**Figure 1:**
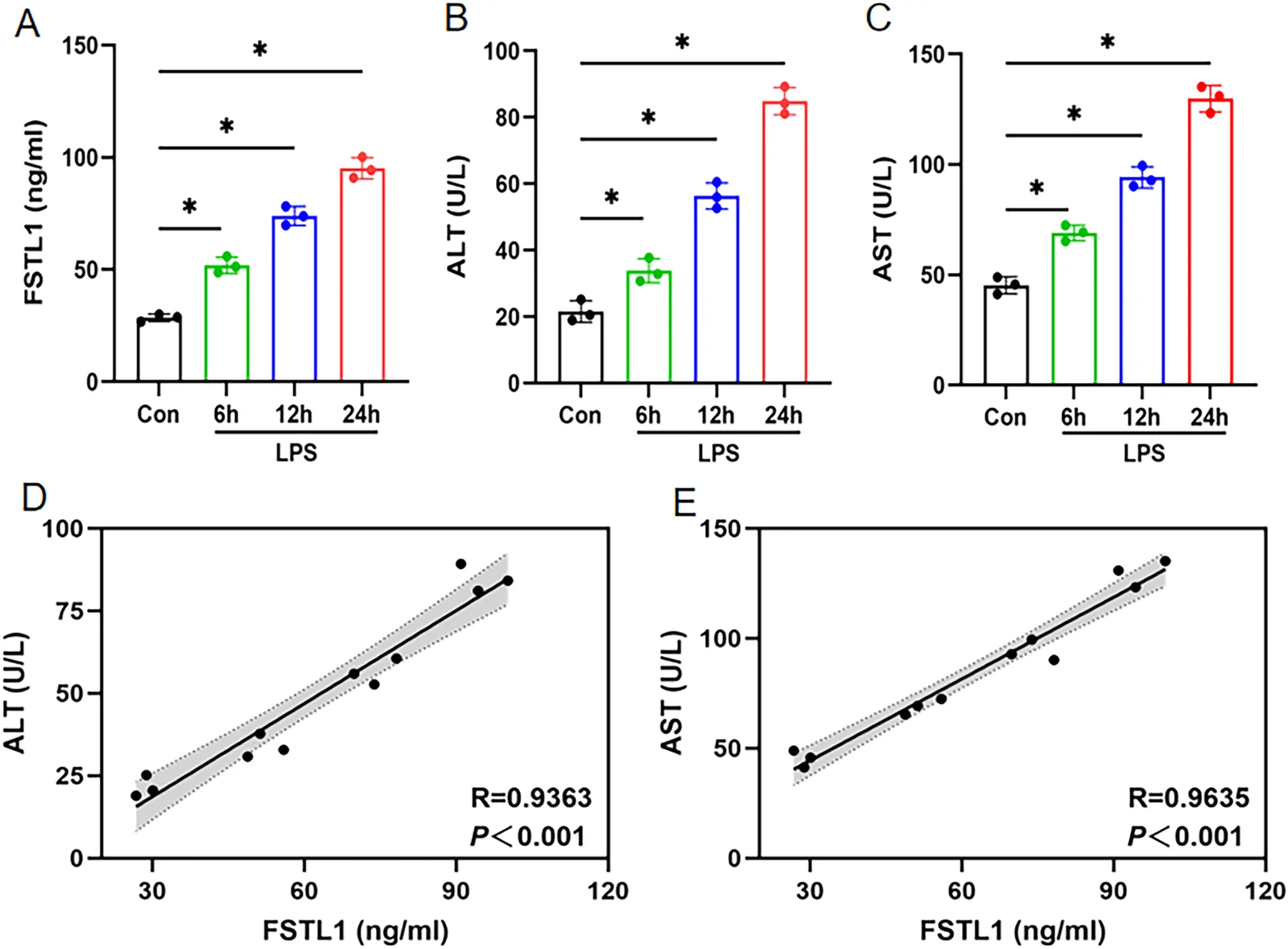
Serum FSTL1 is positively associated with septic liver injury. **(A–C)** The level of FSTL1, ALT, and AST in serum at different time points (0, 6, 12 and 24 h) after LPS treatment (n=3). **(D, E)** Correlation between circulating FSTL1 level and serum ALT and AST levels (n=12). Statistical analyses were performed using one-way ANOVA **(A–C)** and Spearman’s correlation coefficient r with two-tailed P-value **(D, E)**. Data are presented as the mean ± SD *P < 0.05, compared to Con group.

### Treatment with LPS upregulates hepatocyte FSTL1 expression and aggravates liver injury

In the above experiment, we separated the liver tissue for follow-up experiments. Western blot analysis showed that FSTL1 protein expression significantly increased at 6, 12, and 24 h after LPS treatment compared with the control group (**Figure 2A, B**). Meanwhile, we detected the production of proinflammatory cytokines (IL-6 and TNF-α) in liver homogenates. The results indicated that the levels of IL-6 and TNF-α in liver tissue gradually increased as sepsis developed (**Figure 2C, D**). The expression of FSTL1 was positively correlated with interleukin-6 (IL-6) and tumor necrosis factor-alpha (TNF-α) levels (**Figure 2E, F**). In addition, the pathological changes of liver tissues were detected through H&E staining. Similarly, liver tissue injury gradually worsened with the development of sepsis, and the expression of FSTL1 was positively associated with the degree of liver injury (**Figures 2G–I**). These data suggested that FSTL1 could be an important contributor to aggravating LPS-induced liver injury.

**Figure 2:**
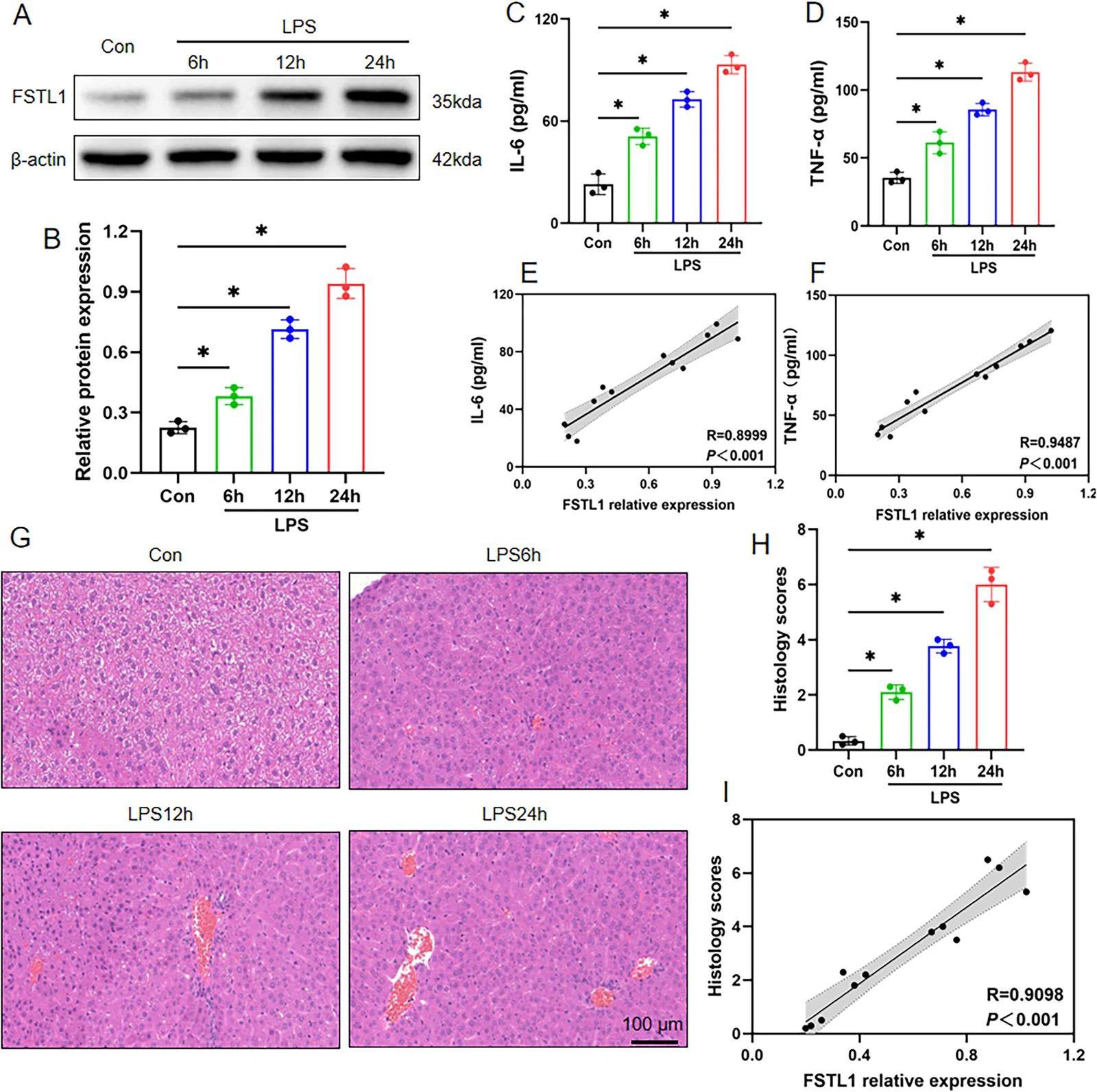
Treatment with LPS upregulates hepatocyte FSTL1 expression and aggravates liver injury. **(A, B)** Representative western blot images and quantification showing FSTL1 protein levels in hepatocytes at different time points (0, 6, 12 and 24 h) after LPS treatment (n=3). **(C, D)** The level of IL-6 and TNF-α in liver homogenates at different time points (0, 6, 12 and 24 h) after LPS treatment (n=3). **(E, F)** Correlation between FSTL1 relative expression in hepatocyte and the levels of IL-6 and TNF-α in liver homogenates (n=12). **(G, H)** Representative images and quantification of HE indicating pathological alterations in liver at different time points (0, 6, 12 and 24 h) after LPS treatment (n=3). I, Correlation between FSTL1 relative expression in hepatocyte and liver injury. Statistical analyses were performed using one-way ANOVA **(A–D, G, H)** and Spearman’s correlation coefficient r with two-tailed P-value **(E, F, I)**. Data are presented as the mean ± SD *P < 0.05, compared to Con group.

### Treatment with LPS induces the activation of the NLRP3-CASP1-GSDMD and BAX-CASP3-GSDME pyroptosis pathways in hepatocytes

Besides, we also examined the activation of NLRP3-CASP1-GSDMD and BAX-CASP3-GSDME pyroptosis pathways in liver tissue. Western blot analysis showed that compared with the control group, the protein levels of NLRP3, CASP1, GSDMD, BAX, CASP3, and GSDME were gradually upregulated at 6, 12, and 24 h after LPS stimulation (**Figures 3A–G**). Flow cytometry analysis revealed that the numbers of hepatocellular death also gradually increased at 6, 12, and 24 h after LPS stimulation (**Figure 3H, I**). We also measured the concentration of IL-1β, IL-18, lactate dehydrogenase (LDH), and high mobility group box-1 protein (HMGB1) in liver homogenates and noted that they were elevated at 6, 12, and 24 h after LPS (**Figure 3J, M**). These results suggested that the NLRP3-CASP1-GSDMD and BAX-CASP3-GSDME pyroptosis pathways were activated after sepsis.

**Figure 3:**
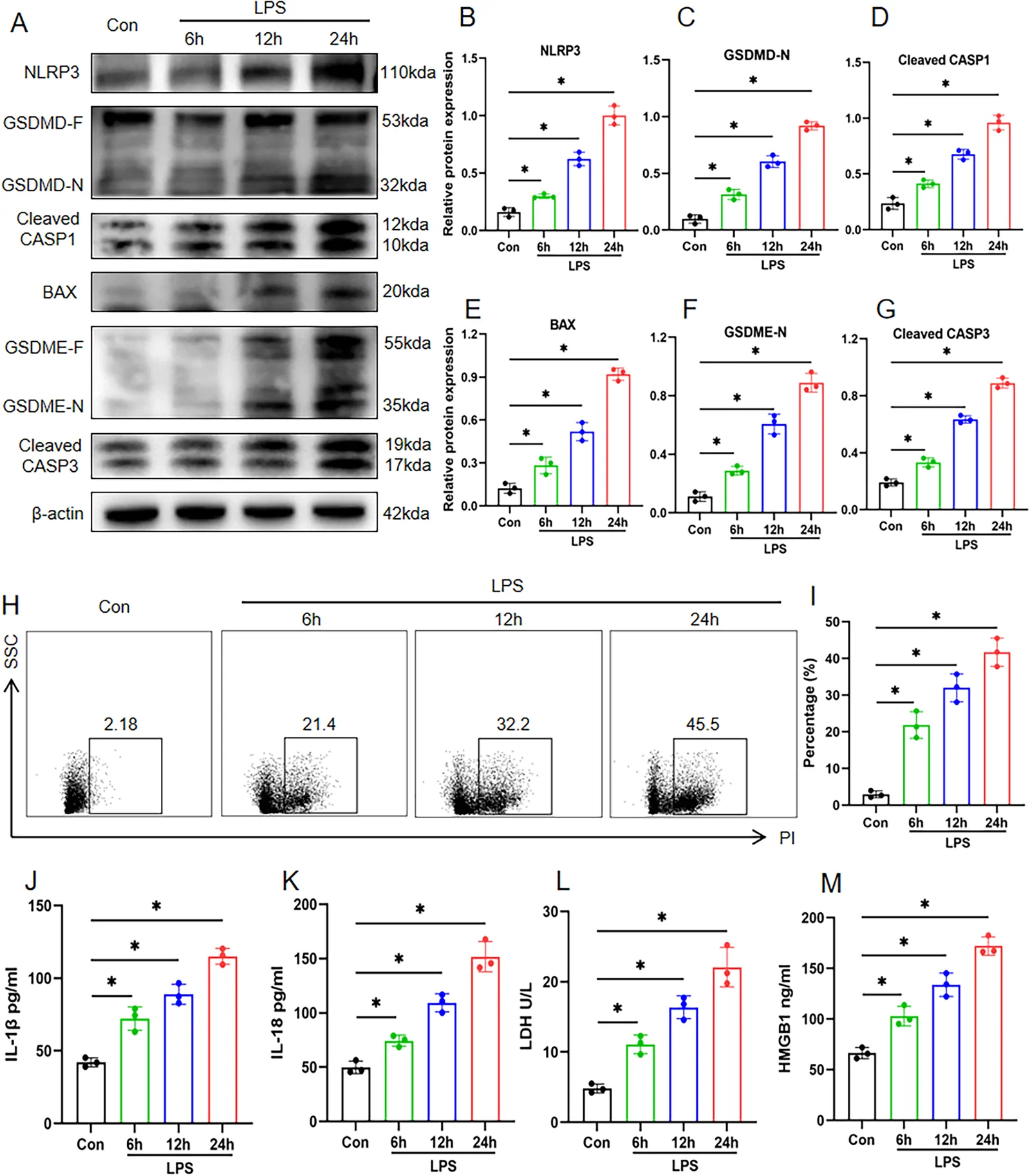
Treatment with LPS induces the activation of the NLRP3-CASP1-GSDMD and BAX-CASP3-GSDME pyroptosis pathways in hepatocytes. **(A–G)** Representative western blot images and quantification showing pyroptosis-related protein levels in hepatocyte at different time points (0, 6, 12 and 24 h) after LPS treatment (n=3). **(H, I)** Representative flow cytometry plots with quantitative bar charts showing the hepatocyte death rate (n=3). **(J–M)** The level of IL-1β, IL-18, LDH and HMGB1 in liver homogenates at different time points (0, 6, 12 and 24 h) after LPS treatment (n=3). Statistical analyses were performed using one-way ANOVA **(A–M)**. Data are presented as the mean ± SD *P < 0.05, compared to Con group.

### Establishment of liver-specific FSTL1 knockdown mice

To further explore the role of FSTL1 in septic liver injury, we utilized an AAV8 construct to knock down FSTL1 expression in a hepatocyte-specific manner in mice. The mice were injected with liver-specific AAV8 vectors (AAV8-TBG-ShFSTL1 or AAV8-TBG-ShCtrl) via the tail vein. Two weeks after AAV injection, the knockdown efficiency of FSTL1 in the liver was determined in the ShCtrl and ShFSTL1 groups (**Figure 4A**). As expected, the expression of FSTL1 was significantly reduced in the liver of ShFSTL1 mice compared to ShCtrl controls, with no significant changes in other tissues (kidney, heart, lung, spleen, or intestine) (**Figure 4B, C**).

**Figure 4:**
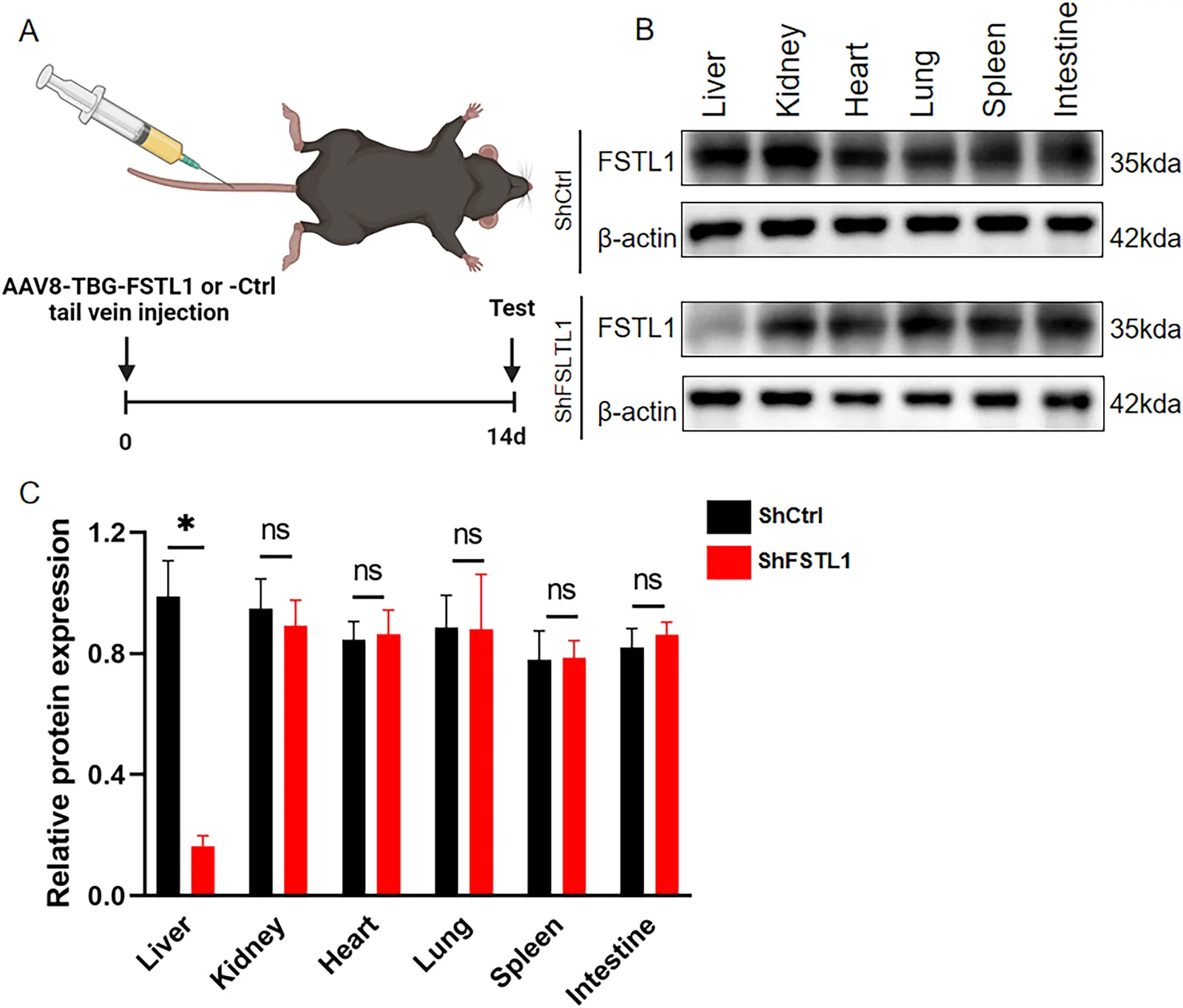
Establishment of liver-specific FSTL1 knockdown mice. **(A)** Schematic workflow showing generation of conditional knockdown mice in which FSTL1 was specifically ablated in hepatocytes by using AAV8. **(B, C)** Western blot analysis of FSTL1 in different organs in ShCtrl and ShFSTL1 mice (n=3). One-way ANOVA was used for in statistical analyses **(B, C)**. Data are presented as mean ± SD. *P < 0.05 vs. the ShCtrl group; ns, not significant.

### FSTL1 deficiency attenuates LPS-induced NLRP3-CASP1-GSDMD and BAX-CASP3-GSDME pyroptosis pathways activation

Next, we assessed the impact of FSTL1 on sepsis by using ShCtrl and ShFSTL1 mice. Western blot analysis showed that liver-specific knockdown of FSTL1 significantly inhibited the expression of NLRP3, CASP1, GSDMD, BAX, CASP3, and GSDME (**Figure 5A, G**). Flow cytometry analysis indicated that compared with mice in the ShCtrl + LPS group, hepatocellular death was obviously diminished in the ShFSTL1 + LPS group (**Figure 5H, I**). Meanwhile, LPS-induced release of pyroptosis-related cytokines and damage-associated molecular patterns (DAMPs), including IL-1β, IL-18, LDH, and HMGB1, was suppressed by interfering with FSTL1 expression (**Figure 5J, M**). These results indicated that loss of FSTL1 could significantly inhibit LPS-induced inflammatory pyroptosis pathway activation.

**Figure 5:**
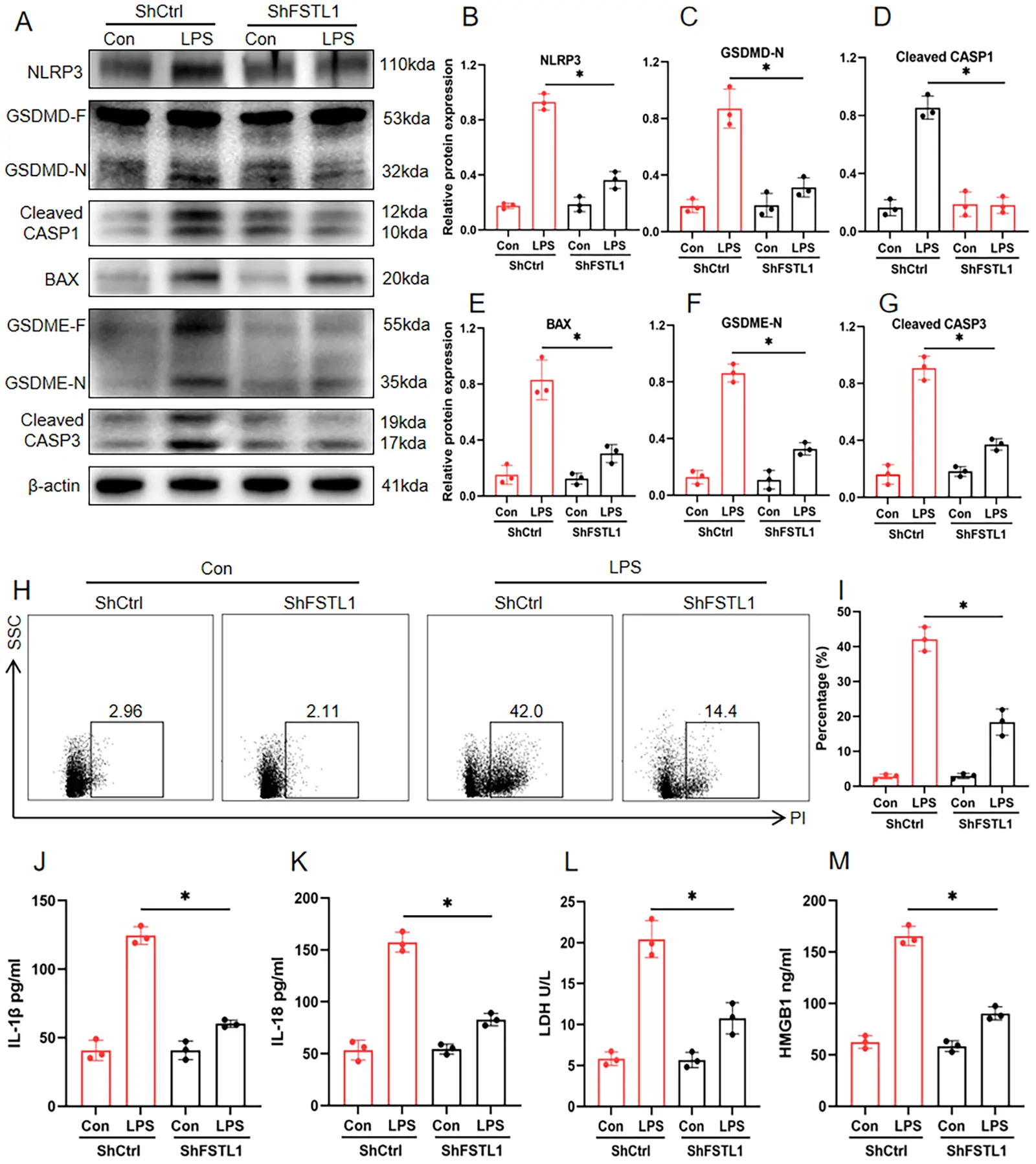
FSTL1 deficiency attenuates LPS-induced NLRP3-CASP1-GSDMD and BAX-CASP3-GSDME pyroptosis pathways activation. **(A–I)** After treatment with PBS or LPS for 24 h, hepatocytes were isolated from ShCtrl and ShFSTL1 mice and used for further assays. **(A–G)** Representative western blot images and quantification showing pyroptosis-related protein levels in hepatocyte (n=3). **(H, I)** Representative flow cytometry plots with quantitative bar charts showing the hepatocyte death rate (n=3). **(J–M)** The level of IL-1β, IL-18, LDH and HMGB1 in liver homogenates from each group (n=3). Statistical analyses were performed using one-way ANOVA **(A–M)**. Data are presented as the mean ± SD *P < 0.05, compared to ShCtrl + LPS group.

### FSTL1 deficiency alleviates LPS-induced liver injury and improves the survival rate of septic mice

As shown in **Figure 6**, conditional knockdown of FSTL1 in hepatocytes contributed to an obvious improvement in liver injury (**Figure 6A, B**). Meanwhile, LPS-induced production of IL-6 and TNF-α was also decreased in liver homogenates (**Figure 6C, D**). Besides, compared with mice in the ShCtrl + LPS group, ShFSTL1 + LPS group mice showed a significant reduction in mortality (**Figure 6E**). These results indicated that loss of FSTL1 could alleviate septic liver injury and increase survival rate of mice.

**Figure 6:**
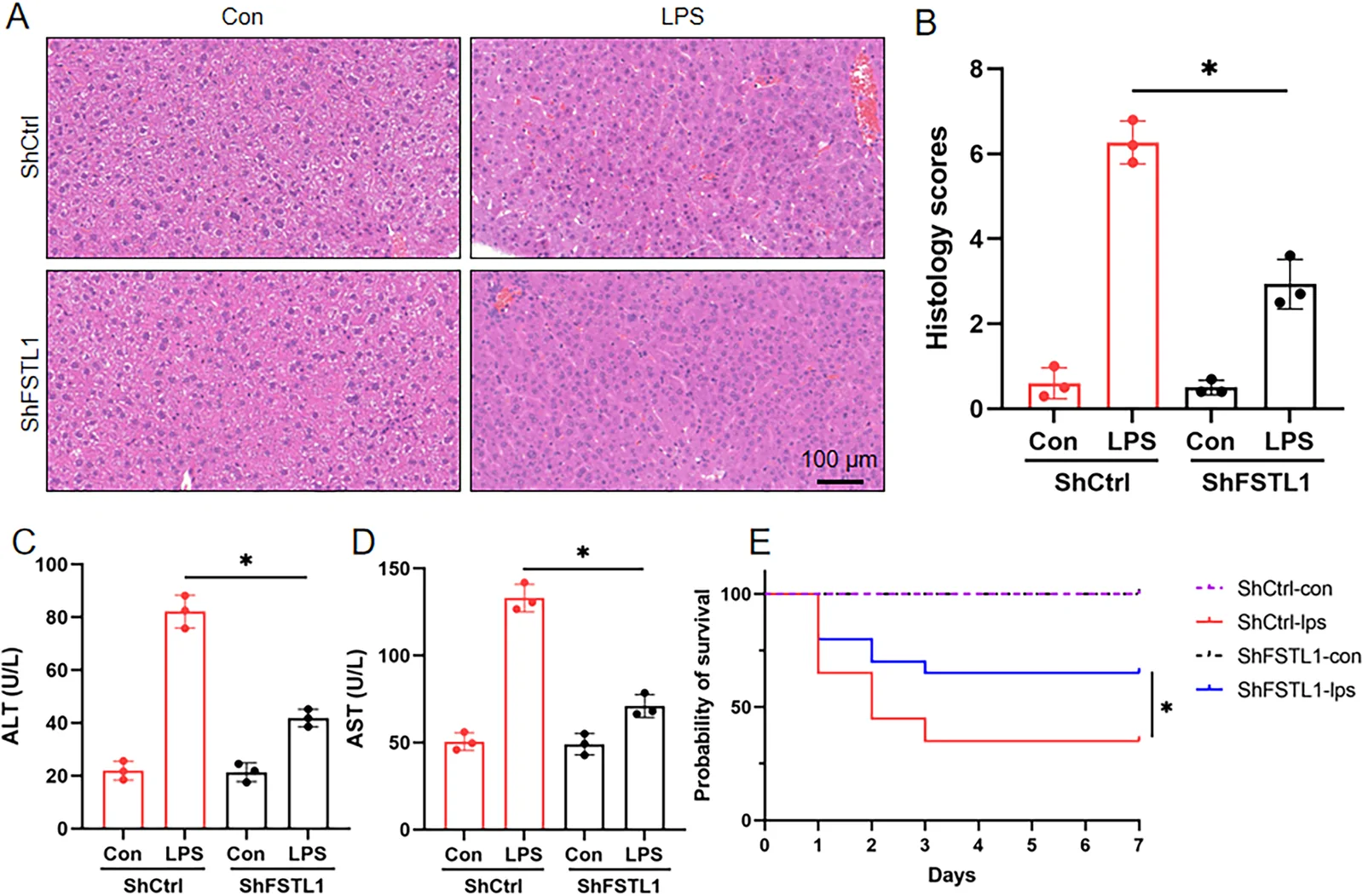
FSTL1 deficiency alleviates LPS-induced liver injury and improves the survival rate of septic mice. **(A, B)** Representative images and quantification of HE indicating pathological alterations in liver from each group (n=3). **(C, D)** The level of ALT, and AST in serum from each group (n=3). Survival analysis of mice in each group (n=20). Statistical analyses were performed using one-way ANOVA **(A–D)**. Statistical analysis in **(E)** was conducted using survival curve comparison with the log-rank test. Data are presented as the mean ± SD *P < 0.05, compared to ShCtrl + LPS group.

### FSTL1 deficiency reduces hepatocyte ROS production

It has been proved that oxidative stress caused by ROS accumulation is an important mechanism underlying cell death, and growing evidence has demonstrated that ROS is a crucial activator for various cell death pathways, including apoptosis, pyroptosis, and ferroptosis (26–28). To investigate the effect of ROS on the two pyroptotic pathways in hepatocytes, we isolated hepatocytes for *in vitro* experiments and administered the ROS scavenger NAC before LPS stimulation. The results showed that, compared with the LPS group, ROS levels were significantly reduced in the LPS+NAC group (**Figure 7A, B**). Meanwhile, western blot, ELISA, and flow cytometry results all indicated that NAC significantly inhibited the activation of the two pyroptotic signaling pathways, thereby attenuating hepatocyte pyroptosis (**Figures 7C–G**). Additionally, in the above animal experiment, flow cytometric analysis showed that ROS levels were significantly lower in the ShFSTL1 + LPS group than in the ShCtrl + LPS group (**Figure 7J, K**). Consistently, immunofluorescence staining revealed weaker fluorescence intensity in the ShFSTL1 + LPS group than in the ShCtrl + LPS group (**Figure 7L**). These findings suggest that FSTL1-mediated regulation of pyroptosis pathway activation is associated with ROS signaling.

**Figure 7:**
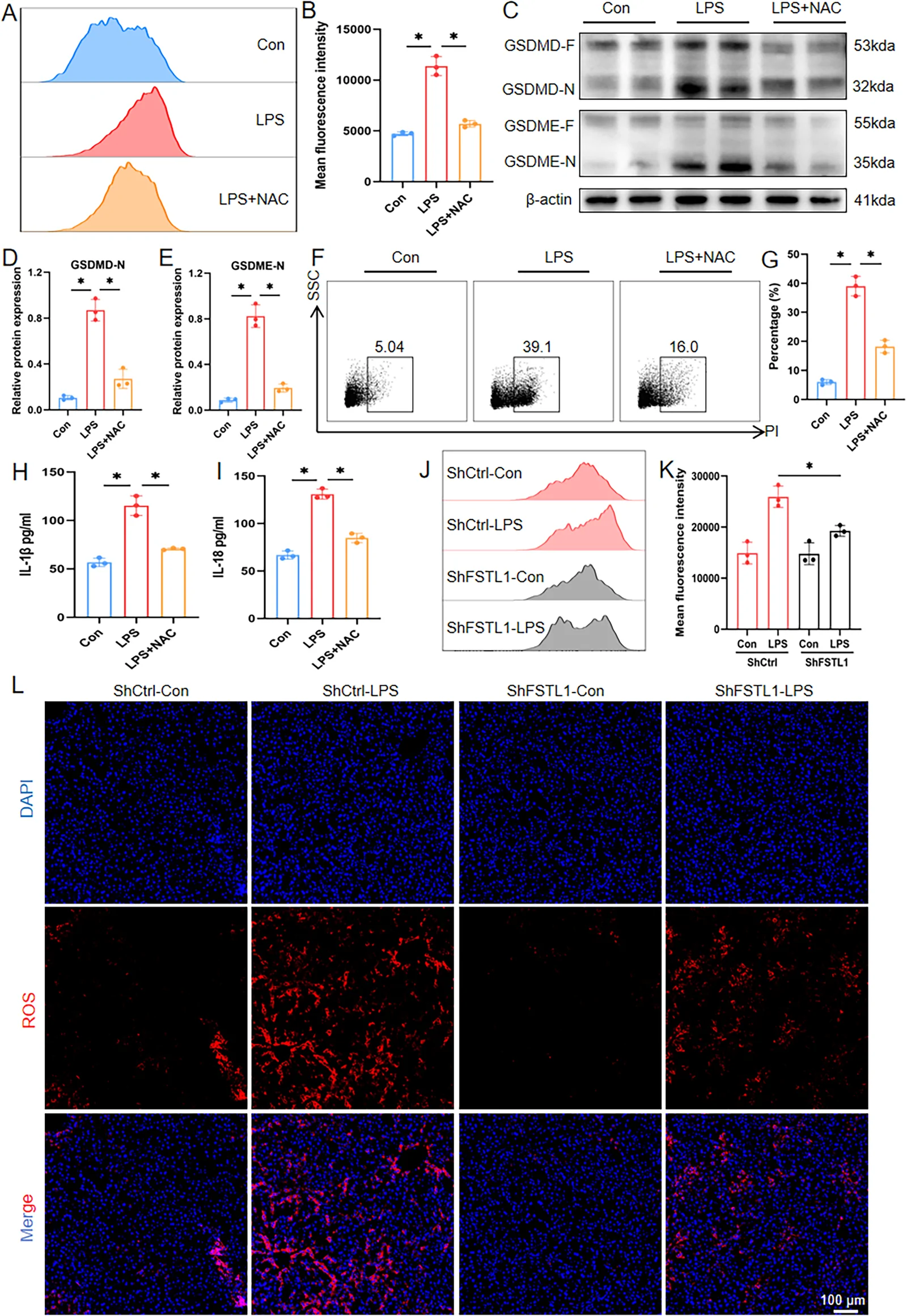
FSTL1 deficiency reduces hepatocyte ROS production. **(A–I)** Isolated hepatocytes were treated with LPS (100 ng/mL) or PBS (vehicle control) in the presence or absence of 10 mM NAC for 24 h. **(A, B)** Histogram and quantitative bar charts displaying the levels of ROS in hepatocytes, as measured by flow (n=3). **(C–E)** Representative western blot images and quantification showing pyroptosis-related protein levels in hepatocyte (n=3). **(F, G)** Representative flow cytometry plots with quantitative bar charts showing the hepatocyte death rate (n=3). **(H, I)** The levels of IL-1β and IL-18 in the culture supernatants of hepatocytes (n = 3). **(J–L)** After treatment with PBS or LPS for 24 h, hepatocytes were isolated from ShCtrl and ShFSTL1 mice and used for further assays. **(J, K)** Histogram and quantitative bar charts displaying the levels of ROS in hepatocytes, as measured by flow (n=3). **(L)** Representative immunofluorescence images showing ROS generation in hepatocytes. Statistical analyses were performed using one-way ANOVA **(A, B)**. Data are presented as the mean ± SD *P < 0.05, compared to ShCtrl + LPS group.

## Discussion

In this study, we found that serum FSTL1 level and hepatocellular FSTL1 expression were gradually increased during sepsis and their levels were positively correlated with LPS-induced liver injury. Similarly, sepsis induced gradual upregulation of pyroptotic pathways in hepatocytes, involving both the NLRP3-CASP1-GSDMD and the BAX-CASP3-GSDME pathways. Loss of FSTL1 in hepatocytes significantly inhibited the activation of NLRP3-CASP1-GSDMD and BAX-CASP3-GSDME pyroptosis, and FSTL1 deficiency could markedly alleviate septic liver injury and increase survival rate of septic mice. Subsequent experiments demonstrated that FSTL1 knockdown lowered hepatocyte ROS levels. Combined with our NAC-based ROS-scavenging assays, these observations support that ROS is linked to FSTL1-associated pyroptosis.

Growing evidence has confirmed that FSTL1 plays a proinflammatory role in various diseases, and its functional activation can lead to uncontrolled inflammation and aggravated organ injury (29). Cheng et al. found that FSTL1 accelerates the development of liver fibrosis by promoting M1 polarization, upregulating proinflammatory mediator expression, and aggravating macrophage/neutrophil infiltration (30). It was reported that FSTL1 can promote the activation of NLRP3/IL-1β signaling in ovalbumin (OVA)-induced asthma mouse models to exacerbate airway inflammatory response and cytokine release (23). Another study confirmed that FSTL1 is critically involved in the development of osteonecrosis of the femoral head by promoting nuclear factor κB (NF-κB) signaling pathway activation and inducing inflammatory cytokine production (31). Serum levels of FSTL1 have been found to be positively correlated with non-alcoholic steatohepatitis progression in humans (32). Notably, research has demonstrated that plasma FSTL1 levels are significantly elevated in sepsis patients, with even higher concentrations observed in non-survivors compared to survivors (33). Nevertheless, the exact mechanism by which FSTL1 contributes to sepsis pathogenesis remains poorly understood. In this study, we found that elevated FSTL1 levels in serum and liver tissue correlated with sepsis severity. Conversely, hepatocyte-specific FSTL1 knockdown conferred protection against LPS-induced liver injury and enhanced survival in septic mice. Our data indicated that FSTL1 contributed substantially to the pathological progression of liver damage in sepsis.

Aberrant activation of hepatocyte pyroptosis not only impairs cellular repair capacity, but also further amplifies cell death signaling pathways and worsens injury by releasing DAMPs and inflammatory cytokines (13, 34, 35). Recently, a study found that pharmacological inhibition of GSDMD is insufficient to rescue septic mice from pyroptosis-induced lethal inflammation. The ablation of GSDMD and GSDME can significantly ameliorate pyroptosis-induced inflammatory damage and improve survival rate of septic mice (36). Zeng et al. found that when the NLRP3-CASP1-GSDMD pyroptosis pathway is blocked, adenosine triphosphate (ATP), an activator of pyroptosis, can induce macrophage pyroptosis through the CASP3/GSDME pathway (37). These findings suggest that combined observation of CASP3-GSDME and CASP1-GSDMD pyroptosis pathways may provide new ideas for understanding the pathophysiological process of human diseases. In this study, we observed time-dependent activation of NLRP3-CASP1-GSDMD and BAX-CASP3-GSDME pyroptosis pathways in hepatocytes, which was similar to FSTL1 expression.

Accumulating evidence demonstrates that FSTL1 is a key regulator of multiple programmed cell death pathways, with critical implications for numerous human diseases (29). Recently, a study has found that in Aβ_1-42_-induced Alzheimer’s disease model mice, the ablation of FSTL1 improves neural oscillations by alleviating neuroinflammation and inhibiting apoptosis (38). It was reported that CAF-derived FSTL1 can promote NCOA4-dependent ferroptosis in NK cells, thus resulting in diminished cytotoxic function of NK cells and enhanced gastric cancer progression (24). Another study revealed that FSTL1 can increase the expression of NLRP3 and promote CASP1 activation (33). Similarly, Fang et al. demonstrated that FSTL1 exacerbates silica-induced pulmonary injury in mice by enhancing NLRP3 inflammasome activation in macrophages and subsequent IL-1β secretion (39). In the present study, we found that FSTL1 expression was positively correlated with pyroptotic pathway activation. Genetic interference with FSTL1 expression showed a significant inhibition in the activation of GSDMD- and GSDME-mediated pyroptosis. Besides, it has been confirmed that ROS can induce pyroptosis through GSDMD and GSDME pathways (35). In our study, we observed that hepatocytes exhibited marked ROS accumulation under septic conditions, which could be attenuated by reducing ROS production or suppressing FSTL1 expression.

Nonetheless, our study had some limitations. First, our observations support a link between FSTL1-driven pyroptosis and ROS production; however, the exact mechanism by which FSTL1 regulates ROS generation remains unclear and requires further investigation. Second, we only explored the effects of FSTL1 on septic liver injury, while its potential effects on other organ systems remain to be elucidated. Third, our study was limited to the LPS-induced endotoxemia model, which does not fully recapitulate clinical polymicrobial sepsis; therefore, future validation using the CLP model and clinical studies is warranted. Fourth, we acknowledge that pharmacological inhibition alone cannot fully establish target specificity. Future studies using hepatocyte-specific FSTL1 overexpression, rescue experiments, or genetic ablation of GSDMD/GSDME will be necessary to confirm the specificity of the pathway. Our current conclusion is based on loss-of-function evidence and is therefore associative rather than definitive.

## Conclusion

In summary, this study reveals that FSTL1 exerts pro-pyroptotic effects in hepatocytes and promotes LPS-provoked liver injury, which is correlated with ROS-dependent activation of both NLRP3-CASP1-GSDMD and BAX-CASP3-GSDME pyroptosis pathways. Our findings suggest that FSTL1 may serve as a potential biomarker candidate for septic liver injury surveillance and highlight its therapeutic potential in sepsis.

## Data Availability

The raw data supporting the conclusions of this article will be made available by the authors, without undue reservation.
